# Supplementary material for: Dueling dynamics of low-angle normal fault rupture with splay faulting and off-fault damage
Source: Nat Commun. 2023 Apr 24;14:2352. doi: 10.1038/s41467-023-37063-1 (PMC10126135; doi:10.1038/s41467-023-37063-1)
Supplement: Supplementary file 1 — Supplementary Information [file 41467_2023_37063_MOESM1_ESM.docx]

*Nature Communications*

Supplementary Information for

**Dueling dynamics of low-angle normal fault rupture with splay faulting and off-fault damage**

J. Biemiller^1,*^, A.-A. Gabriel^1,2^, & T. Ulrich^2^

*^1^ Institute of Geophysics and Planetary Physics, Scripps Institution of Oceanography, University of California San Diego, La Jolla, CA, USA*

*^2^ Department of Earth & Environmental Sciences, Ludwig Maximilian University of Munich, Munich, Germany*

** Now at United States Geological Survey, Geology, Minerals, Energy and Geophysics Science Center, Portland, OR, USA*

***Corresponding author: J. Biemiller (***[***jbiemiller@ucsd.edu***](mailto:jbiemiller@ucsd.edu)***)& A.-A. Gabriel (***[***algabriel@ucsd.edu***](mailto:algabriel@ucsd.edu)***)***

**Contents of this file**

Supplementary Figures S1 to S11

Figure S1. Modeled peak slip rates after 10 s (unless otherwise noted) for different homogeneous (left) and heterogeneous (right) values of *L*, the characteristic slip distance. For heterogeneous models, the characteristic slip distance increases from a minimum value of *L_hypo_* within 3 km of the hypocenter to *L_outer_* for points >5 km from the hypocenter. See Text S1 (“Earthquake nucleation & peak slip rates”) for discussion of these results.

Figure S2-4. Fault slip and (clipped) accumulated plastic strain for models with weak (a), intermediate-strength (b), and strong (c) sediments above 4 km depth (identical to those in main text Fig. 4d-f). Modeled plastic strain in each row is plotted on a different color scale, highlighting how the selected color scale can influence the appearance and interpretation of distributed vs. localized coseismic off-fault damage. Plotted on a color scale commonly used for dynamic rupture simulations with off-fault plasticity^1,2^, plastic deformation appears widespread and distributed through the hanging-wall wedge (Fig. S2, top row). In contrast, plastic damage appears localized along narrow fault-like shear bands in the hanging wall (Fig. S4, bottom row) when plotted with lower strain bounds of a similar order of magnitude to those used as thresholds for identifying coseismic damage zones in geodetic studies^3^ and those reported in modern and historical ruptures^4,5,6,7^.

**Figure S5.** Peak slip rates and (clipped) accumulated plastic strain for models with different values of the characteristic slip distance, *L*. These results suggest that different values of *L* and corresponding peak slip rates may influence patterns of coseismic off-fault deformation, with larger *L* and lower peak slip rates resulting in deeper-seated subplanar plastic deformation bands (interpreted as incipient splay faulting) than models with smaller *L* and lower peak slip rates.


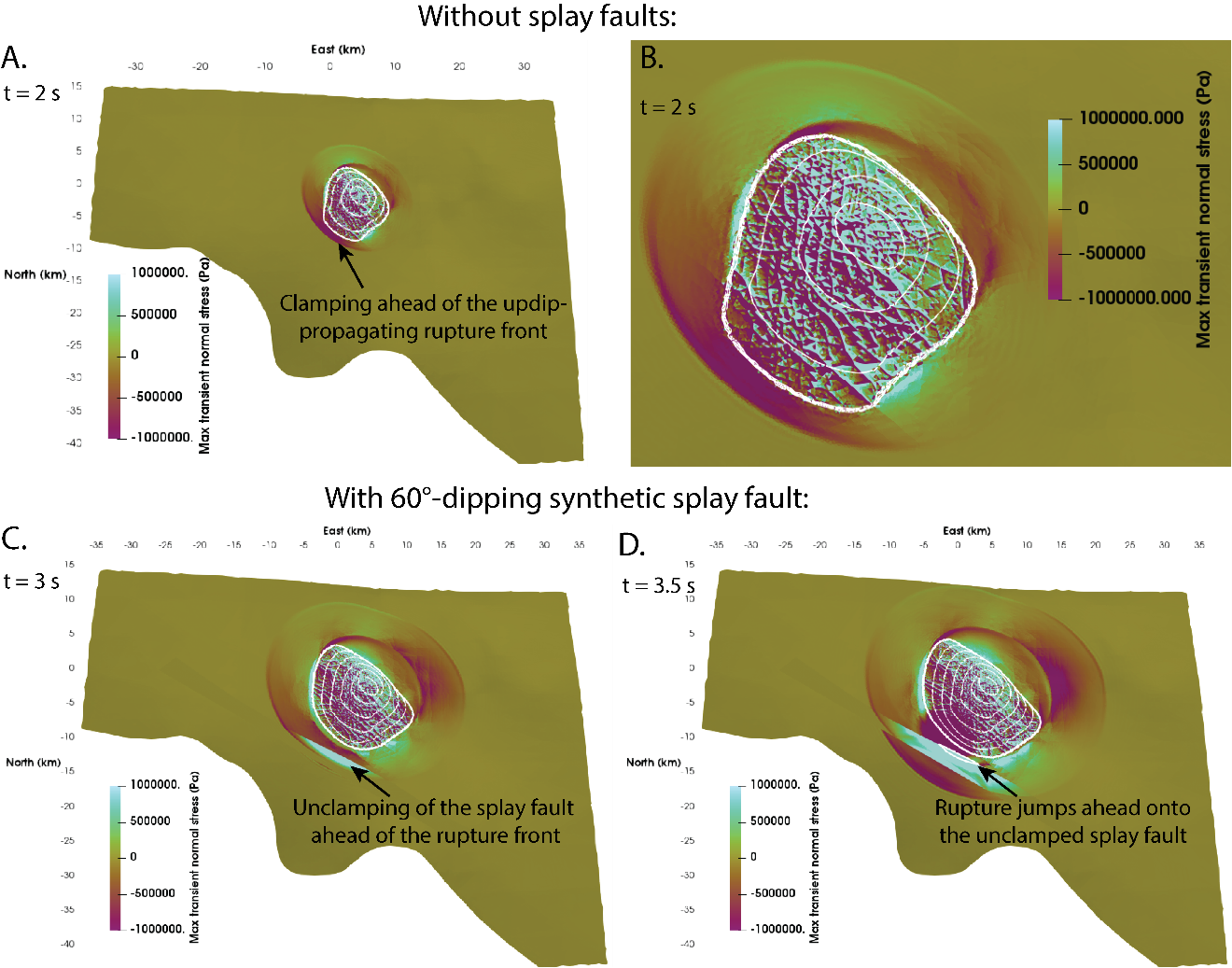


**Figure S6.** A.) Mapview of the entire fault and B.) Enlarged plot of the hypocentral region showing maximum transient normal stress at *t* = 2 s in the reference model without splay faults or plasticity (Figure 3G). White curves show 0.5 s rupture contours. Arcs of elevated negative normal stress indicate increased compression and clamping ahead of the updip-propagating rupture front induced by slip on the deeper ruptured portion of the fault, as predicted by previous dynamic rupture modeling of variably-dipping normal faults^8,9^. C.) Maximum transient normal stress at *t* = 3 s in the model with a synthetic splay fault dipping 60° (Figure 3B). White curves show 0.5 s rupture contours. In contrast to A & B, increased positive normal stress on the deepest portion of the splay indicates tension and unclamping of the splay fault ahead of the rupture front. This unclamping allows rupture to jump ahead onto the splay fault before the rupture front reaches the intersection of the splay and the underlying low-angle normal fault, as shown in D.) at *t* = 3.5 s.


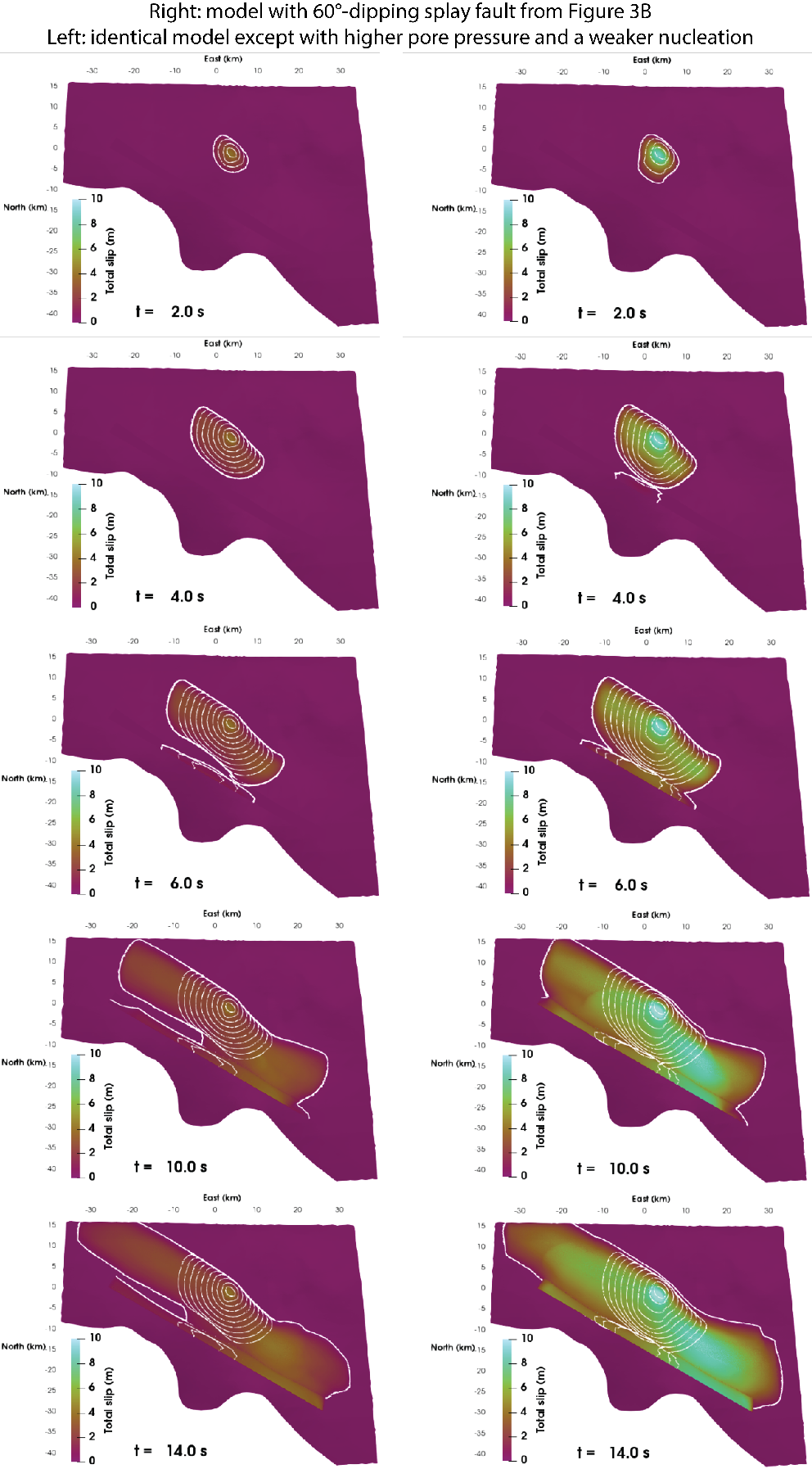


**Figure S7.** Right column: Evolution of total slip with 0.5 s rupture contours for the model with a 60°-dipping synthetic splay fault (Figure 3B); Left column: Evolution of total slip with 0.5 s rupture contours for a model with identical geometry and parameters except with higher pore fluid pressure (*λ_f_* = 0.66) and weaker nucleation stresses (up to 30 MPa) applied over a smaller hypocentral area of radius 2 km. These models illustrate how initial stress conditions affect resulting rupture velocities, and the balance between fracture energy and energy release rate^10^ relate to modeled splay-detachment dynamics. Slow ruptures with less energy at the rupture front induce weaker dynamic stress perturbations than their fast, energetic counterparts. Whether slow rupture or slow slip propagates onto the shallow detachment or slips the splay fault would thus be more sensitive to how favorably or unfavorably oriented for slip each fault is relative to the far-field tectonic stress field, as predicted by static Andersonian-type fault mechanics^11^. Fast ruptures, on the other hand, energetically and rapidly propagate updip and along-strike, transmitting strong dynamic stresses that interact with the free surface as well as neighboring faults. Thus, preferential rupture of the detachment or splay faults would be more strongly influenced by dynamic processes such as rupture interaction with the free surface for fast ruptures than for their slower-rupturing counterparts. Rupture velocity mirrors the available rupture energy, with energetic fast ruptures propagating past structural or rheological slip barriers much more easily than slower ruptures. Processes dissipating rupture energy must act more effectively in order to fully arrest a fast strong rupture.


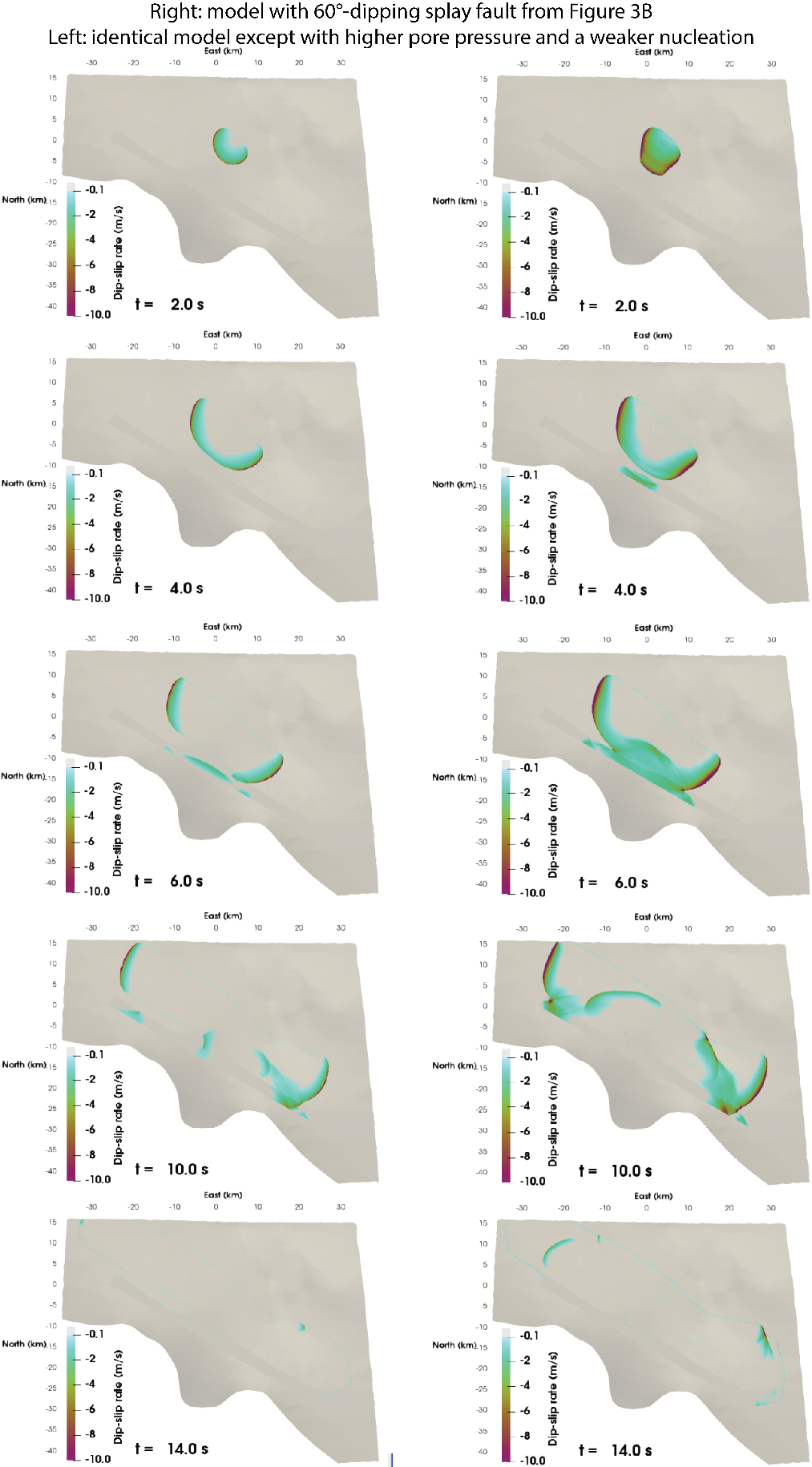


**Figure S8.** Right column: Dip-slip rate for the model with a 60°-dipping synthetic splay fault (Figure 3B); Left column: Dip-slip rate for a model with identical geometry and parameters except with higher pore fluid pressure (*λ_f_* = 0.66) and weaker nucleation stresses (up to 30 MPa) applied over a smaller hypocentral area of radius 2 km.”


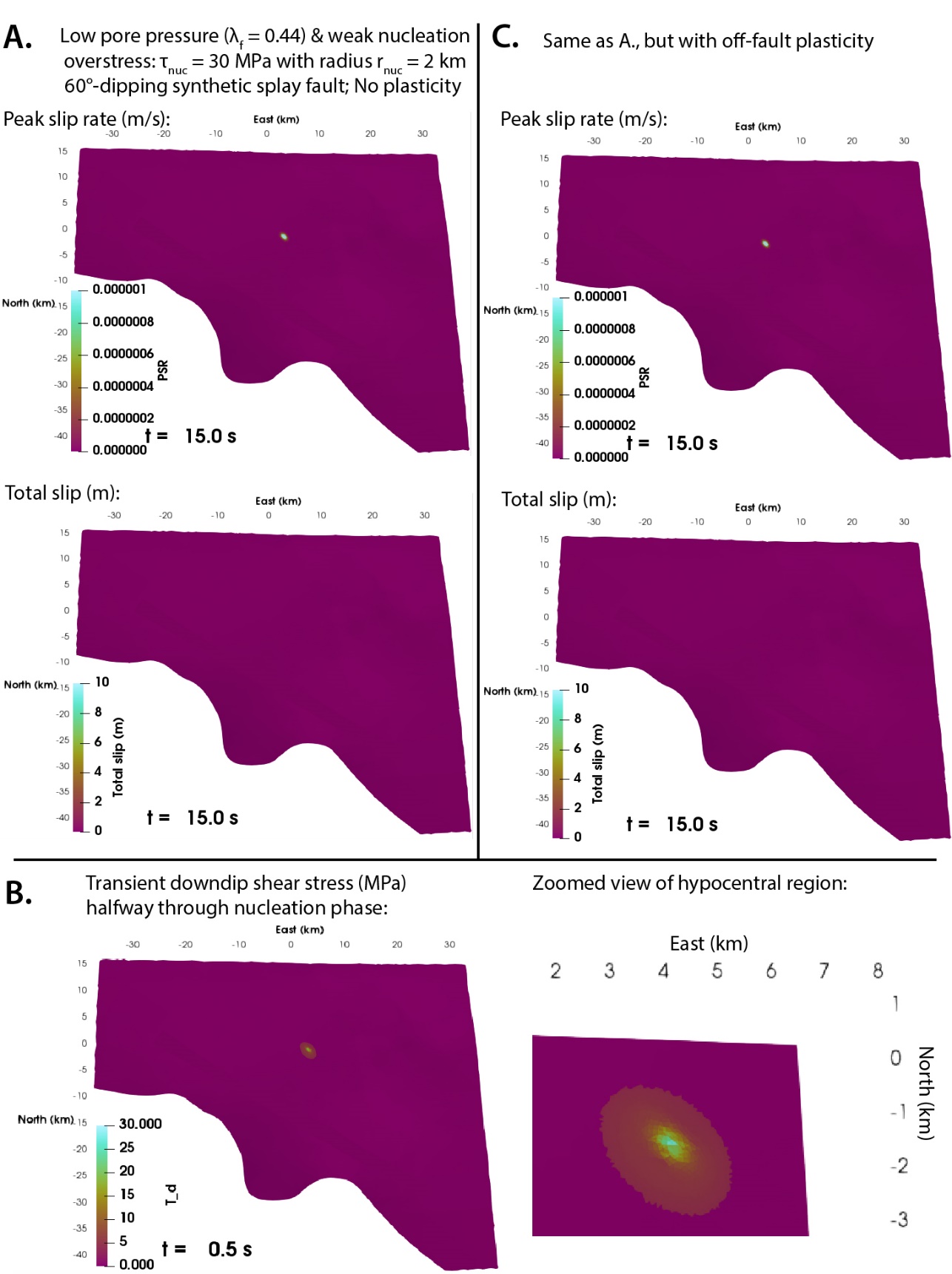


**Figure S9.** A.) Peak slip-rate and total slip for an elastic model with the near-hydrostatic pore fluid pressures (*λ_f_* = 0.44) used throughout this study and the weaker nucleation conditions used in the previous single-fault elastic models^12^, with overstresses up to 30 MPa applied over a smaller hypocentral area of radius 2 km, as shown by the plot of transient downdip shear stress at *t* = 0.5 s in B. These stress conditions fail to nucleate sustained dynamic rupture. C.) Dynamic rupture similarly fails to nucleate in a model identical to that in A and B but with off-fault plasticity enabled.


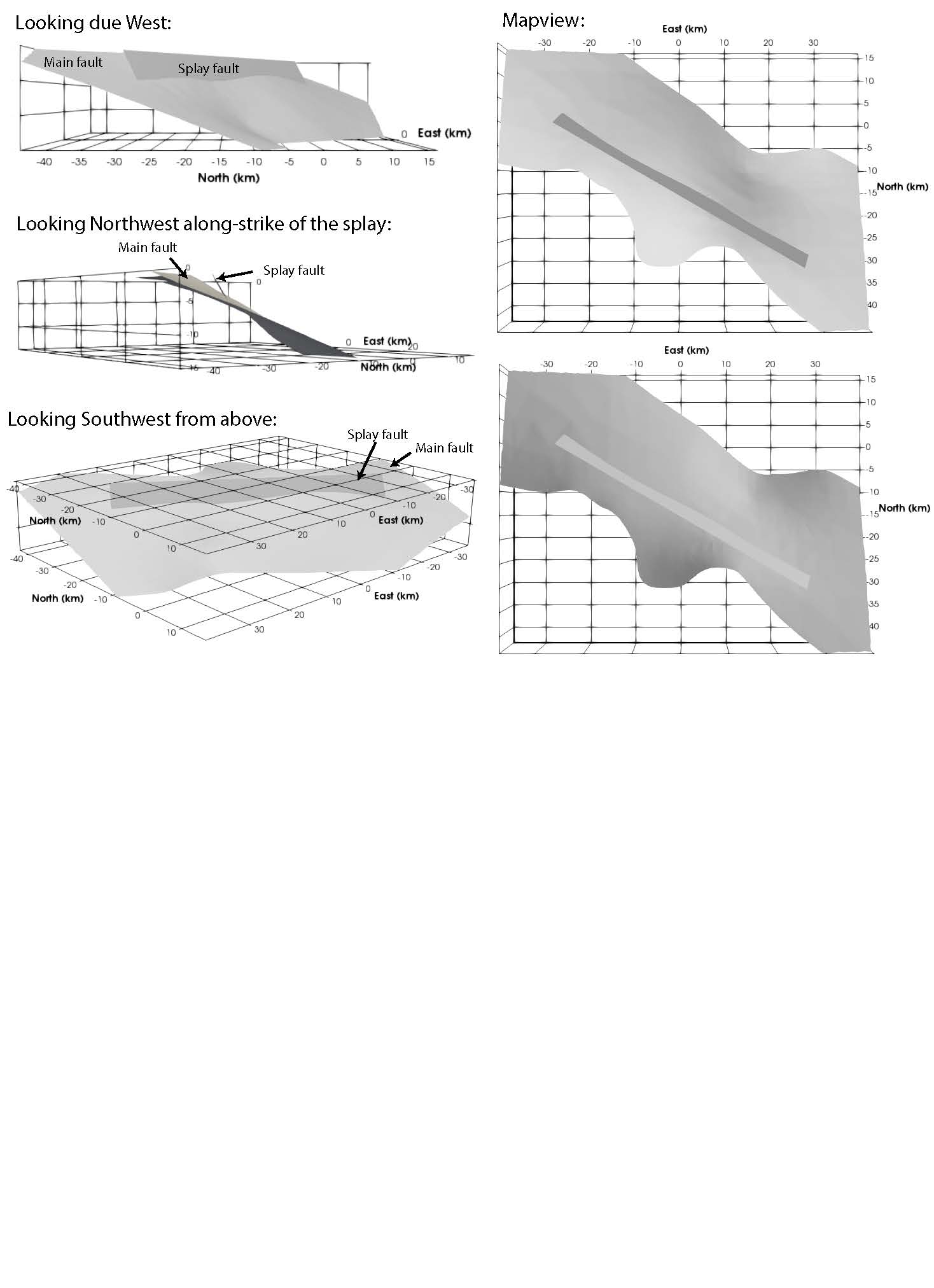


**Figure S10.** Additional plots of the model with a 60°-dipping synthetic splay fault (Figure 3B) illustrating the prescribed fault geometries. Left: different view angles with main and splay fault labeled. Right: modeled faults viewed from above but lit with different lighting options.


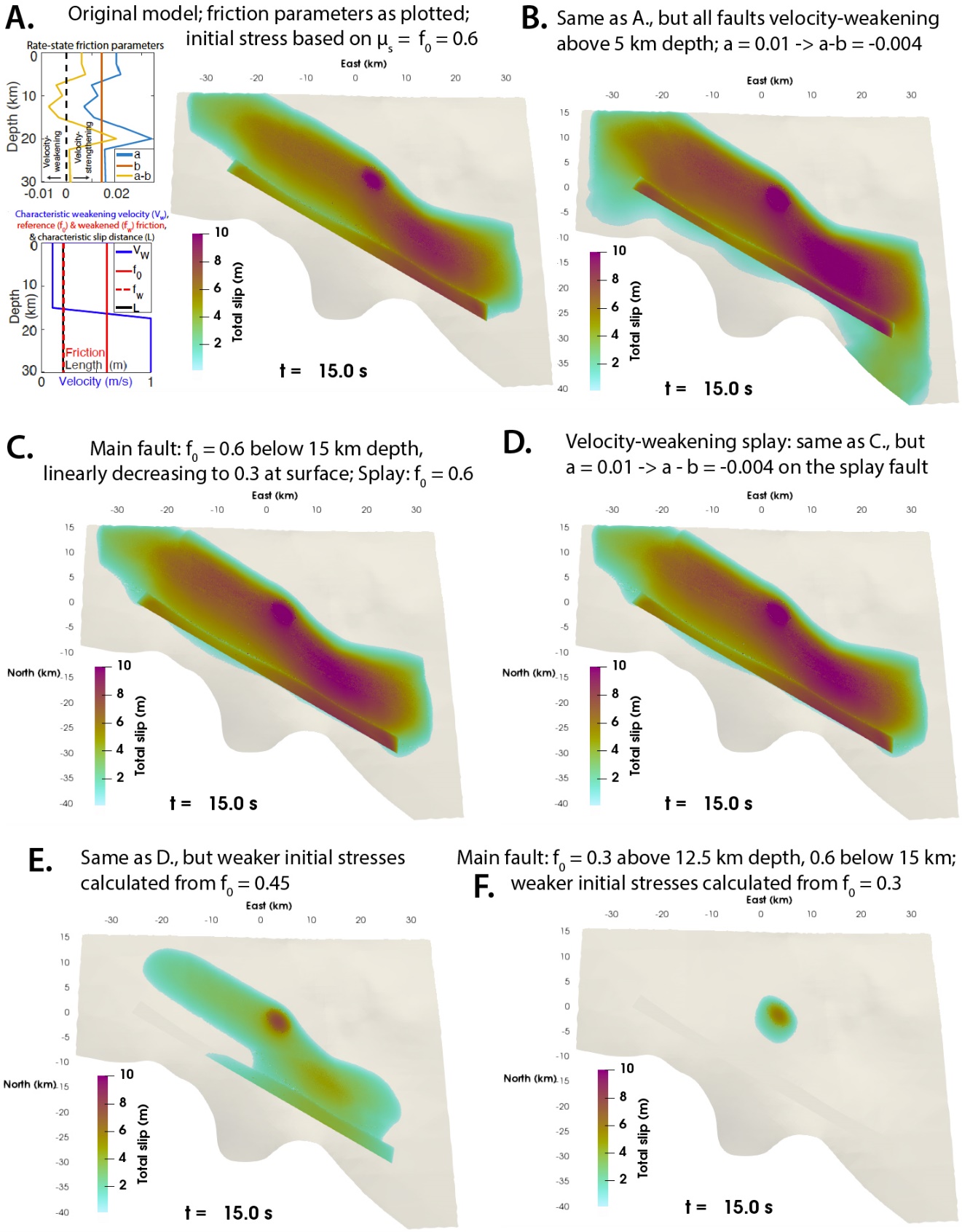


**Figure S11.** Total slip after 15 s for A.) the model with a 60°-dipping synthetic splay fault (Figure 3B) and B-F.) additional variants of that model isolating the effects of variable fault friction and stress. B.) Model with shallow velocity-weakening friction on both faults results in more total slip and allows rupture to penetrate slightly further updip on the main fault, above the fault intersection at 5 km depth, highlighting the stabilizing effects of clay-rich velocity-strengthening gouges in these fault systems. C-E.) Models with friction and stress conditions designed to test the mechanical influence of variable levels of fault maturity between the long-lived, high-offset Mai’iu fault and the younger, immature low-offset splay. C.) A weaker main fault with lower effective static friction decreasing above 15 km depth to *f_0_* = 0.3 at the surface results in similar but muted additional slip than in B, suggesting that the strength of the main fault may be less important than its frictional stability in modulating shallow rupture patterns. D.) Model with identical conditions to C except velocity-weakening materials in the splay fault generates indistinguishable slip patterns from those of C, suggesting splay fault slip is largely insensitive to splay fault frictional stability. E.) Model with lower initial stresses calculated based on a weaker coefficient of static friction of 0.45 results in similar patterns of main and splay fault slip but with significantly less total slip, while F.) one with even weaker stresses and static friction of 0.3 fails to nucleate sustained dynamic rupture. E & F suggest that if fault strength were governed by the weak velocity-strengthening gouges in the mature LANF core, interseismic loading would be relieved by creep and thus interseismic stresses could not accumulate to levels capable of producing the large paleoearthquakes recorded in these LANF systems.

**References for Supplementary Information**

1. Ma, S., & Hirakawa, E. T. (2013). Dynamic wedge failure reveals anomalous energy radiation of shallow subduction earthquakes. *Earth and Planetary Science Letters*, *375*, 113–122. https://doi.org/10.1016/j.epsl.2013.05.016
2. Wilson, A., & Ma, S. (2021). Wedge Plasticity and Fully Coupled Simulations of Dynamic Rupture and Tsunami in the Cascadia Subduction Zone. *Journal of Geophysical Research: Solid Earth*, *126*(7), 1–27. https://doi.org/10.1029/2020JB021627
3. Barnhart, W. D., Gold, R. D., & Hollingsworth, J. (2020). Localized fault-zone dilatancy and surface inelasticity of the 2019 Ridgecrest earthquakes. *Nature Geoscience*, *13*(10), 699–704. https://doi.org/10.1038/s41561-020-0628-8
4. Michel, R., & Avouac, J. P. (2006). Coseismic surface deformation from air photos: The Kickapoo step over in the 1992 Landers rupture. *Journal of Geophysical Research: Solid Earth*, *111*(B3), 3408. https://doi.org/10.1029/2005JB003776
5. Elliott, A. J., Dolan, J. F., & Oglesby, D. D. (2009). Evidence from coseismic slip gradients for dynamic control on rupture propagation and arrest through stepovers. *Journal of Geophysical Research: Solid Earth*, *114*(B2), 2312. https://doi.org/10.1029/2008JB005969
6. Oskin, M. E., Arrowsmith, J. R., Corona, A. H., Elliott, A. J., Fletcher, J. M., Fielding, E. J., … Teran, O. J. (2012). Near-field deformation from the El Mayor-Cucapah earthquake revealed by differential LIDAR. *Science*, *335*(6069), 702–705. https://doi.org/10.1126/SCIENCE.1213778/SUPPL_FILE/702.MP3
7. Gold, P. O., Oskin, M. E., Elliott, A. J., Hinojosa-Corona, A., Taylor, M. H., Kreylos, O., & Cowgill, E. (2013). Coseismic slip variation assessed from terrestrial lidar scans of the El Mayor–Cucapah surface rupture. *Earth and Planetary Science Letters*, *366*, 151–162. https://doi.org/10.1016/J.EPSL.2013.01.040
8. Oglesby, D. D., Archuleta, R. J., & Nielsen, S. B. (1998). Earthquakes on dipping faults: The effects of broken symmetry. *Science*, *280*(5366), 1055–1059. https://doi.org/10.1126/science.280.5366.1055
9. Aochi, H. (2018). Dynamic asymmetry of normal and reverse faults due to constrained depth-dependent stress accumulation. *Geophysical Journal International*, *215*(3), 2134–2143. https://doi.org/10.1093/gji/ggy407
10. Weng, H., & Ampuero, J.-P. (2022). Integrated rupture mechanics for slow slip events and earthquakes. *Nature Communications*, *13*(1), 7327. https://doi.org/10.1038/s41467-022-34927-w
11. Sibson, R. H. (1990). Rupture nucleation on unfavorably oriented faults. *Bulletin of the Seismological Society of America*, *80*(6A), 1580–1604. https://doi.org/10.1785/BSSA08006A1580
12. Biemiller, J., Gabriel, A.-A., Ulrich, T., & Biemiller, J. (2022). The Dynamics of Unlikely Slip: 3D Modeling of Low-angle Normal Fault Rupture at the Mai’iu Fault, Papua New Guinea. *Geochemistry, Geophysics, Geosystems*, e2021GC010298. https://doi.org/10.1029/2021GC010298
